# Evaluation of antioxidant and antiproliferative activity of *Flueggea leucopyrus* Willd (katupila)

**DOI:** 10.1186/1472-6882-14-274

**Published:** 2014-07-30

**Authors:** Preethi Soysa, Irushi Shamalika De Silva, Jayantha Wijayabandara

**Affiliations:** Department of Biochemistry and Molecular Biology, Faculty of Medicine, University of Colombo, Colombo-08, Sri Lanka; Faculty of Medical Sciences, University of Sri Jayewardenapura, Nugegoda, Sri Lanka

**Keywords:** *Flueggea leucopyrus*, Traditional medicine, Anticancer activity, Radical scavenging activity, Apoptosis

## Abstract

**Background:**

*Flueggea leucopyrus* Willd is a shrub grown in many parts of the dry zones in Sri Lanka. The leaves of *F. leucopyrus* has been used for treating cancer in the traditional system of medicine in Sri Lanka. Hence, this study was performed to analyze the antioxidant and antiproliferative properties of the aqueous extract of the leaves of *F. leucopyrus* on *HEp-2* cells.

**Method:**

The aqueous extract of *F. leucopyrus* leaves (AEFLL) was freeze dried. Total phenolic content was assayed using Folin Ciocalteu reagent. Antioxidant activities of the extracts were evaluated using *in vitro* assays: inhibition of DPPH (1,1-diphenyl-2-picrylhydrazyl) radical scavenging and 2-deoxy-D-ribose degradation assay. Nitric oxide radical scavenging activity was determined by using Griess reagent. The MTT, LDH assays and protein synthesis were used to study antiproliferative and cytotoxic activities against the *Hep-2* cell after 24 hour exposure. DNA fragmentation and microscopic examination of cells stained with a mixture of ethidium bromide/acridine orange were used to visualize apoptosis in *HEp-2* cells treated with the AEFLL.

**Results:**

The total phenolic content of the extract was 22.15 ± 1.65 (w/w) % of gallic acid equivalent. The values for EC_50_ were 11.16 ± 0.37, 4.82 ± 1.82 and 23.77 ± 3.16 μg/mL for DPPH radical scavenging, nitric oxide radical scavenging activity and 2-deoxy-D-ribose degradation assay respectively. The EC_50_ with MTT and LDH assays were 506.8 ± 63.16 and 254.52 ± 42.92 μg/mL respectively. A dose dependent decrease in protein synthesis in *HEp-2* cells was shown with an EC_50_ value of 305.84 ± 12.40 μg/mL. DNA fragmentation and ethidium bromide/acridine assays showed that the AEFLL induces apoptosis in *HEp*-2 cells. These results were in conformity with the morphological changes observed in the cells treated with the AEFLL. The brine shrimp bioassay showed that the AEFLL had no lethality over the concentration range of 50–500 μg/mL.

**Conclusions:**

Aqueous extract of the leaves of *F. leucopyrus* extract demonstrated antioxidant activity *in vitro*. Further it showed antiproliferative properties and induced apoptosis in *HEp-2* cells.

## Background

Reactive oxygen species (ROS) and reactive nitrogen species (RNS) are constantly produced in the body and are neutralized and eliminated by many endogenous mechanisms. However, imbalance of neutralization of such products leads to the oxidative damage of DNA, lipids and proteins [1]. Thus over production of ROS and RNS leads to cancer, cardiovascular disease, atherosclerosis, hypertension, ischemia/reperfusion injury, diabetes mellitus, neurodegenerative diseases, autoimmune, rheumatoid arthritis, and ageing [1, 2]. The evidence from epidemiological and laboratory studies have demonstrated that some edible plants as a whole, or their nutraceutical ingredients with antioxidant properties such as polyphenols have substantial protective effects on human carcinogenesis [3, 4].

*F. leucopyrus* Willd (Katupila) belongs to the family Phyllanthaceae. The plant is found in many parts of Sri Lanka particularly in dry zones as shrubs [5]. The leaves of *F. leucopyrus* have been used in the treatment of cancer, boils, external ulcers and sores in traditional medicine in Sri Lanka. Various species of the genus *Flueggea* are used to treat many diseases including epilepsy, malaria, jaundice, intestinal worms, edema, heavy menstruation, sterility, poliomyelitis and aplastic anemia in many African and Asian countries [6]. More than ten secondary metabolites are identified in *F.leucopyrus* [7, 8]. One of the major active constituent found in methanol–water (80:20) extract of the *F.leucopyrus* leaves is bergenin and it has shown antioxidant and immunomodulatory activities *in vitro* (8). Furthermore bergenin, isolated from *F. microcarpa* leaves has shown lipid lowering activity in hyperlipidaemic rats and also antifungal activity against plant pathogenic fungi [9, 10]. Methanol extract of *F. virosa* leaves contain antiplasmodial activity against *Plasmodium falciparum* and bergenin isolated from the aerial parts of this species has shown anti-arrhythmic effects in rat [11, 12]. Monkodkaew *et al.* [13] reported that betulinic acid, has a higher antiproliferative activity against K562 cells compared to friedelin, epifriedelanol, stigmasterol present in the leaves and twigs of *F. viros*a [13]. Further two dimeric indolizidine alkaloids extracted from roots of *F virosa*, have shown growth inhibitory activity against MCF-7 and MDA-MB-231 human breast and P-388 cell lines [14, 15].

In Sri Lanka, the leaves of *F. leucopyrus* are used in the diet in the form of a salad or `porridge’ in villages and the fruits are consumed in India and Africa [16, 17]. Recently *F. leucopyrus* has become very popular among the people in Sri Lanka, after realizing its therapeutic efficacy. As a result it has become a common practice among cancer patients to use the decoction as a dietary substituent in addition to anticancer treatments. This study was carried out to rationalize the scientific basis of the ethnomedical use of *F. leucopyrus* leaves in cancer therapy.

## Method

### Chemicals and equipment

Chemicals – Folin ciocalteu reagent, chemicals needed for cell culture and cytotoxicity studies were purchased from Sigma –Aldrich (P.O. box 14 508, St Louis, MO 63178 USA). 1-Diphenyl-2-picrylhydrazyl (DPPH), Triton X-100 solution (1%), gallic acid, sulfanilamide and ortho-phosphoric acid were purchased from Fluka (Fluka chemie GmbH, CH – 9471 Buchs). Tris base was purchased from Promega (Promega Corporation, Madison, WI 53711–5399, USA). All chemicals used were of analytical grade.

Shimadzu UV 1601 UV visible spectrophotometer (Shimadzu Corporation, Kyoto, Japan) was used to measure the absorbance. LFT 600 EC freeze dryer was used to obtain the freeze –dried residue of the AEFLL (LFT 600 EC, −90-95°C temperature, Hitachi pump with 10 valves). Cells were incubated at 37°C in a humidified CO_2_ incubator (SHEL LAB/Sheldon manufacturing Inc. Cornelius, OR 97113, USA). Olympus (1X70-S1F2) inverted fluorescence microscope (Olympus Optical Co. Ltd. Japan) for observation of cells and photographs were taken using a Nikon D700 camera (Nikon D700, Japan). Deionized water from LABCONCO (waterproplus) UV ultra filtered water system (LABCONCO Corporation, Kansas city, Missouri 64132–2696) or distilled water was used in all experiments.

### Plant material

The fresh plant material of *F. leucopyrus* (katupila) collected from Gampaha district, Sri Lanka was identified and confirmed by the Department of Botany, Bandaranayake Memorial Ayurveda Research Institute, Nawinna, Sri Lanka and the voucher specimens are deposited at the same Institute.

### Preparation of the extract

The branches bearing the leaves of *F. leucopyrus* were washed with distilled water and dried under indirect sunlight. The dry leaves were ground in a domestic grinder until a fine powder was obtained. The powder was extracted with distilled water (250 g/l) under reflux for 2–3 hours. The crude aqueous extract obtained was filtered through cotton wool and Whatmann filter paper (No. 1), and freeze dried.

### Determination of total phenolic content and antioxidant activity

The total phenolic content of the crude lyophilized sample (n = 3) of the AEFLL was determined using the Folin ciocalteu method [18]. Radical scavenging activity by 1, 1- diphenyl-2-picrylhydrazyl (DPPH), nitric oxide scavenging activity and nonsite-specific hydroxyl radical mediated 2- deoxy-D-ribose degradation were determined according to the methods described previously [19]. The percentage inhibition (%I) was calculated from the following equation: % I = [(absorbance of control – absorbance of sample)/absorbance of control] x 100 %. The effective concentration of the sample required to scavenge the respective radical by 50% (EC_50_) was calculated using the linear segment of the curve obtained with%I against concentration.

### Brine shrimp bioassay

Brine shrimp bioassay is a general bioassay which is indicative of cytotoxicity, various pharmacological actions and pesticidal effects [20]. Extracts with EC_50_ ≤ 30 μg/mL are considered to be cytotoxic [20]. The AEFLL (50-500 μg/mL) diluted with hatching medium was subjected to standard procedure for the brine shrimp assay in a 24 well plate (n = 10 live shrimps/well). The live healthy larvae with constant motion were counted after 24 hours. The percentage lethality was determined by comparing the mean surviving larvae of the test and the control. The percentage of hatch inhibition was calculated as: Number of live nauplii in the test/Number of live nauplii in the control.

### Cell lines and cell culture

*HEp-2* cells were routinely cultured in EMEM containing 10% heat inactivated fetal bovine serum and 1% penicillin/streptomycin. Cells were maintained at 37°C in humidified carbon dioxide incubator.

The cells were grown in DMEM supplemented with 10% heat inactivated fetal bovine serum (FBS), 3% glutamine, sodium bicarbonate and antibiotic (penicillin/streptomycin). The cells were incubated at 37°C in a humidified CO_2_ incubator at all times. The cells (2 × 10^5^ cells/well) were seeded in 24-well plates and incubated overnight with 2 mL of the medium described above to obtain a 70% confluent layer. The monolayer was treated with different concentrations of the plant extract and incubated for 24 hours at 37°C. In all experiments a negative control and a positive control were maintained. Negative control contained only growth media while the positive control contained camptothecin (5 mM, 20 μl).

### MTT assay

Metabolically active cells reduce MTT (3, 4, 5-(dimethylthiazol-2-yl) 2-5-diphenyl tetrazolium bromide) to its purple colored formazan product and MTT assay was used to determine cytotoxicty of the AEFLL.

The cells were treated with different concentrations of the extract and incubated for 24 hours at 37°C as described above. The culture medium was replaced with fresh medium and MTT assay was performed [21]. The purple color product was measured at 570 nm. Percentage cell viability = [1-Absorbance of treated cells/Absorbance of untreated cells]*100. The net absorbance from the wells of the untreated cells (negative control) was taken as the 100% viability. Positive control was performed with camptothecin (5 mM, 20 μl).

### Lactate dehydrogenase (LDH) activity

Lactate dehydrogenase is a cytosolic enzyme, which is released in to the surrounding culture medium upon cell lysis and used to assay cytotoxicity. The lactate dehydrogenase assay was performed to determine the rate of reduction of pyruvate to lactate by the enzyme Lactate Dehydrogenase [22]. The NADH that remained in the mixture was used to calculate the enzyme activity. The cells were treated with different concentrations of the AEFLL and incubated for 24 hours as described previously. The LDH activity of the cell lysate and the culture supernatant of the cells which were treated with the plant extracts, were measured according to manufacturer’s instructions (Randox LDH assay kit). Negative control and positive control with camptothecin (5 mM, 20 μl) were also carried out along with the experiment to measure the LDH leakage. The absorbance was measured at 340 nm at intervals of 15 seconds for 1.5 minutes using an air blank. The rate of decline in NADH (gradient) concentration was used to calculate the LDH activity in the supernatant and the lysate.


The total LDH activity is equal to the sum of the LDH activity obtained for the culture supernatant and cell lysate.

### Cell morphology

The morphological changes of the cells were observed after treatment with different concentrations of the plant extract over 24 hours as previously described. The changes were compared with the positive and negative controls.

### Ethidium bromide and acridine orange staining

Ethidium bromide and acridine orange staining was carried out to determine the induction of apoptosis by AEFLL. Acridine orange (AO) permeates both live and dead cells, stains DNA and makes the nucleus appear green while ethidium bromide (EB) is only taken up by cells with damaged cell membranes [23]. Thus, live cells will be uniformly stained green, apoptotic cells will be stained as orange or displayed orange fragments, when observed under fluorescence microscope depending on the degree of loss of membrane integrity due to co-staining with ethidium bromide.

Cells were seeded in 24 well plates and the confluent layer was treated with the AEFLL at different concentrations for 24 hours at 37°C as described previously. The adherent cells were washed with 1.0 mL of PBS and then removed by adding 1 mL of trypsin-EDTA solution. The supernatant was removed after centrifugation and the cell pellet was resuspended in 25 μl of PBS and 2 μl of the dye mixture containing ethidium bromide (100 mg/mL) and acridine orange (100 mg/mL). A 10 μl aliquot of stained cell suspension was placed on microscope slides, covered with glass slips, and examined immediately under the florescence microscope. Images were photographed using a Nikon D700 camera or digital imaging system connected to microscope.

### DNA fragmentation

Cells were seeded in culture flasks (25 mL) and the confluent layer was treated with the AEFLL at different concentrations for 24 hours at 37°C as described previously. Cells were lysed with 5 mL of lysis buffer (10 mM Tris–HCl, 5 mM EDTA, 200 mM NaCl, 0.2% SDS) and incubated at 37°C for 5 minutes. The contents were centrifuged and the pellet was washed with ice cold SE buffer (5 mL). The pellet was then resuspended with ice cold SE (75 mM NaCl; 25 mM Na_2_ EDTA; pH 8.0) buffer (5 mL) with 10% SDS (500 μl) and proteinase K (25 μl) and incubated at 56°C for 1 hour. A volume of 2 mL of NaCl (5 M) was then added to the mixture and incubated on ice for 5 minutes to precipitate proteins. Cells were then centrifuged for 15 minutes at 10,000 rpm and the supernatant was transferred to a fresh tube. Two volumes of ethanol were added to precipitate the DNA, and the sample was centrifuged for 10 minutes at 10,000 rpm. The supernatants were then discarded, and the pellets were washed with 70% cold ethanol. DNA was dissolved in 15 μl of TE (10 mM tris, pH 8.0 and 1 mM EDTA) buffer and subjected to agarose gel (1.5%) electrophoresis for 2 hours. Finally, the gel was photographed using a UVI pro gel documentation system (UVItec UK) following ethidium bromide staining to determine DNA fragmentation.

### Calculations and statistics

All the results of the experiments were expressed as the mean ± standard deviation (Mean ± SD). The measurements were performed in triplicate and values shown are representative for at least three independent experiments. Least square linear regression analysis was applied using Microsoft excel to determine the EC_50_ values and for the calibration curves. R^2^ > 0.99 was considered as linear for the calibration curves. The linear segment of the percentage inhibition/cytotoxictiy – concentration curve was used to calculate the EC_50_ in each experiment.

## Results

### Total phenolic content

The yield of the lyophilized sample of the AEFLL was 6.56% (w/w) and its phenolic content was 22.15 ± 1.65% of Gallic acid equivalents (GAE).

### Antioxidant activity

The effective concentration of the AEFLL required to scavenge DPPH radicals by 50% (EC_50_) was 11.16 ± 0.45 μg/mL (Table 1). L- Ascorbic acid was used as the positive control to compare the EC_50_ values of the test samples. However the value was higher than that of ascorbic acid which was 4.28 ± 0.32 μg/mL.

**Table 1:** **EC**_**50**_ **values for DPPH, NO and hydroxyl radical scavenging activity of the AEFLL (n = 3) and respective positive controls**

Radical scavenging assay	EC_50_(μg/mL)		
	Plant extract	Ascorbic acid	Gallic acid
DPPH	11.16 ± 0.45	4.28 ± 0.32	-
NO	4.82 ± 1.82	54.26 ± 6.54	-
Hydroxyl radical mediated 2- deoxy-D-ribose degradation	23.77 ± 3.87	-	8.27 ± 0.53

Nitrite generated by sodium nitroprusside was reduced by the AEFLL in a dose dependent manner. Gradual increases in NO inhibition was seen at very low concentrations (0.24-3.9 μg/mL) of the plant extract. It was also observed that the concentration reaches a maximum and was maintained at 60% inhibition over concentrations higher than 30.0 μg/mL. The EC_50_ value of AEFLL was 4.82 ± 1.82 μg/mL (Table 1) showing very high nitric oxide scavenging ability compared to the positive control, ascorbic acid (54.26 μg/mL).

The concentration of the AEFLL required to scavenge the reactive hydroxyl radicals by 50% (EC_50_) evaluated by non site-specific hydroxyl radical mediated 2- deoxy-D-ribose degradation was 23.77 ± 3.87 μg/mL (Table 1). The value was greater than the positive control, gallic acid (8.27 μg/mL).

### Cytotoxicity and apoptosis assays

#### Brine shrimp bioassay

No lethality towards the brine shrimp was observed after 24 hour exposure to the AEFLL within the concentration range of 50- 500 μg/mL.

### MTT assay

**Figure 1:**
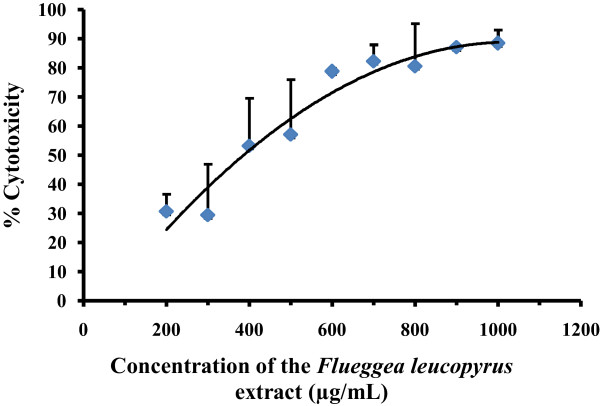
**The percentage cytotoxicity on** ***HEp-2*** **cell line as determined by MTT assay, after 24 hour treatment with the AEFLL.** The data are presented as mean ± SD of three independent experiments. The linear segment of the dose response curve was used to determine EC_50_ value.

The cell viability after 24 hour treatment with the AEFLL was determined by MTT reduction assay. A dose response curve for the percentage of viable cells was obtained against the concentration (Figure 1). The EC_50_ value obtained for the mean of the four independent sample preparations was 506.80 ± 72.93 μg/mL (Table 2). Positive control (camptothecin) showed 76.07 ± 1.72% growth inhibition at the concentration (5 mM, 20 μL) used.

**Table 2:** **The EC**_**50**_ **values obtained for MTT, LDH and brine shrimp assays for AEFLL and Percentage inhibition of cell growth by camptothecin (5 mM, 20 μL)**

Cytotoxicity assay	EC_50_value (μg/mL)	% inhibition by camptothecin (5 mM, 20 μL)
MTT (n = 4)	506.80 ± 72.93	76.07 ± 1.72%
LDH (n = 4)	254.52 ± 42.92	50.51 ± 7.67%
Brine shrimp (n = 3)	>500	

### LDH leakage assay

**Figure 2:**
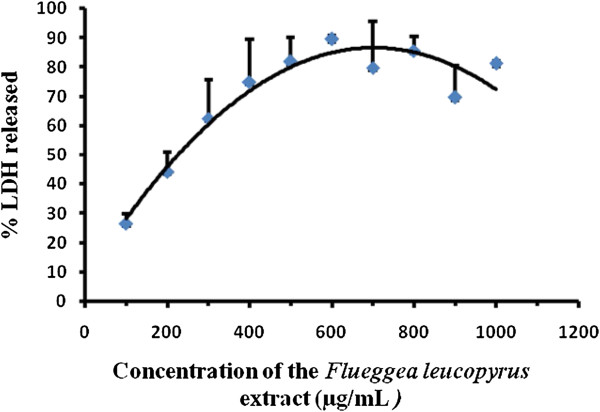
**The percentage LDH released after 24 hour treatment with the AEFLL on** ***HEp-2*** **cell line.** The data are presented as mean ± SD of four independent experiments. The linear segment of the dose response curve was used to determine EC_50_ value.

A dose dependent increase in LDH release to the culture media was observed at concentrations up to 600 μg/mL and a decline of LDH release was shown over 700 μg/mL (Figure 2). Further it was observed that there was a decrease of enzyme activity in culture media as well as in the lysate at higher concentrations. The mean EC_50_ of the percentage cytotoxicity over 24 hour exposure to the plant extract was 254.52 ± 42.92 μg/mL (Table 2). The percentage LDH found in the supernatant of negative control and the positive control with camptothecin were 24.62 ± 6.21 and 50.51 ± 7.67% respectively. The data indicate that, compared to the negative control, there is no significant increase (p > 0.05) in LDH release at a concentration of 100 μg/mL.

### Morphological changes

**Figure 3:**
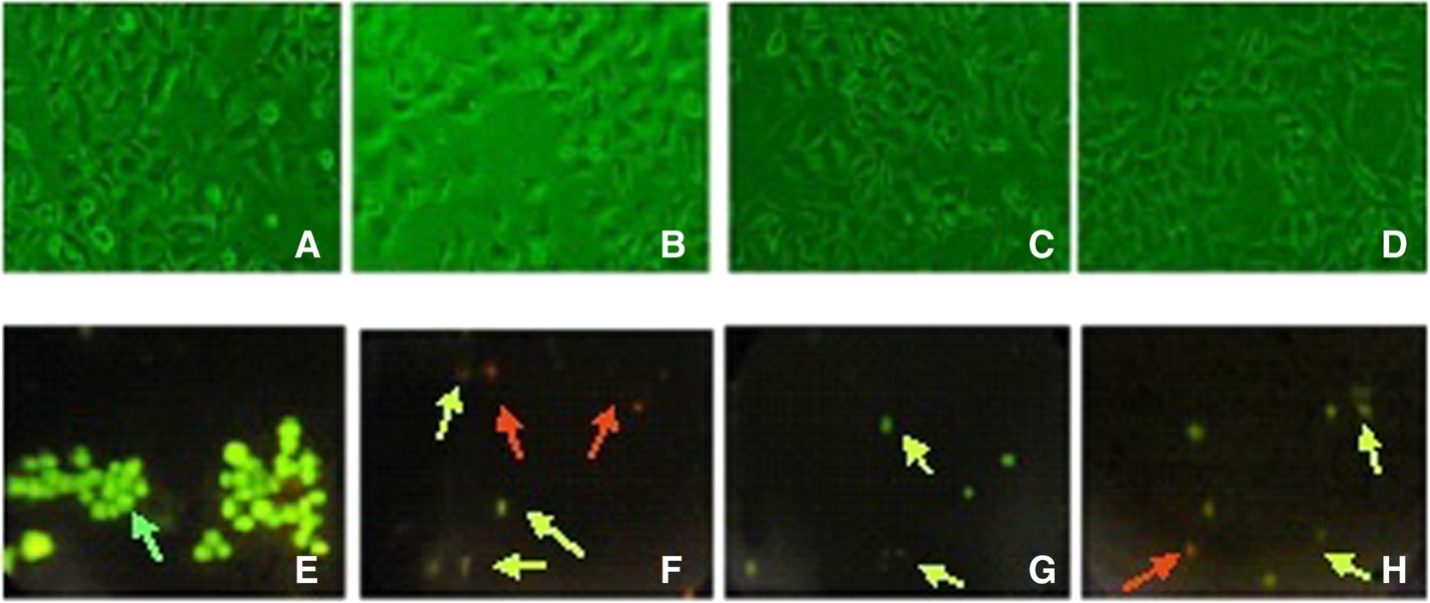
**Light micrographs of** ***HEp-2*** **cell line after 24 hours of incubation with the AEFL at different concentrations.** Cells treated **A**-negative Control; **B**-Positive Control (Camptothecin); **C**- 100 μg/mL; **D** - 300 μg/mL. Live cells have definite morphology and dead cell are rounded. Reduction in cell density also observed in positive control and the AEFL treated cells. Acridine orange-ethidium bromide (AO/EB) fluorescent staining detection of apoptotic morphology in *Hep2* cells treated with the AEFL at different concentrations are depicted in the bottom row. **E**-negative control; **F**- Camptothecin as the positive control (5 mM; 25 μL); **G**- 400 μg/mL, **H** - 800 μg/mL. This figure represents the results of at least 3 independent experiments. Green arrows: live cells, greenish yellow: apoptotic cells (some cells are fragmented and faded color), orange red: late apoptotic cells. (Original magnification 40×).

The cytotoxic effects of the AEFLL on *HEp-2* cells were analyzed using inverted fluorescence microscope and the images are presented in Figures 3. The untreated cells (negative control) shows elongated cells that adhered to the culture plate in comparison with cells that were treated with camptothecin (positive control) where the cells became oval or irregular in shape with highly condensed contents. The *HEp-2* cells which were treated with different concentrations of AEFLL extract showed a dose dependent increase in cell death with increasing concentrations of the drug in comparison to the negative control. The highest concentration of the AEFLL (1000 μg/mL) displayed the rounding, detachment and of cell death as indicated in Figure 3 (C and D).

### Ethidium bromide/acridine orange staining

The microscopic examination of cell morphology of drug treated cells following 24 hour incubation showed characteristics of cell death which were then further investigated using ethidium bromide/acridine orange staining method to determine whether the growth inhibitory activity of the leaf extract was related to the induction of apoptosis (Figure 3). On the basis of the staining procedure live cells with normal nuclei presented bright green nuclei, early apoptotic cells showed green nuclei and late apoptotic cells displayed condensed orange nuclei. In addition dying cells (green but fading of colour) and decreasing of cell density were also observed.

### DNA fragmentation

**Figure 4:**
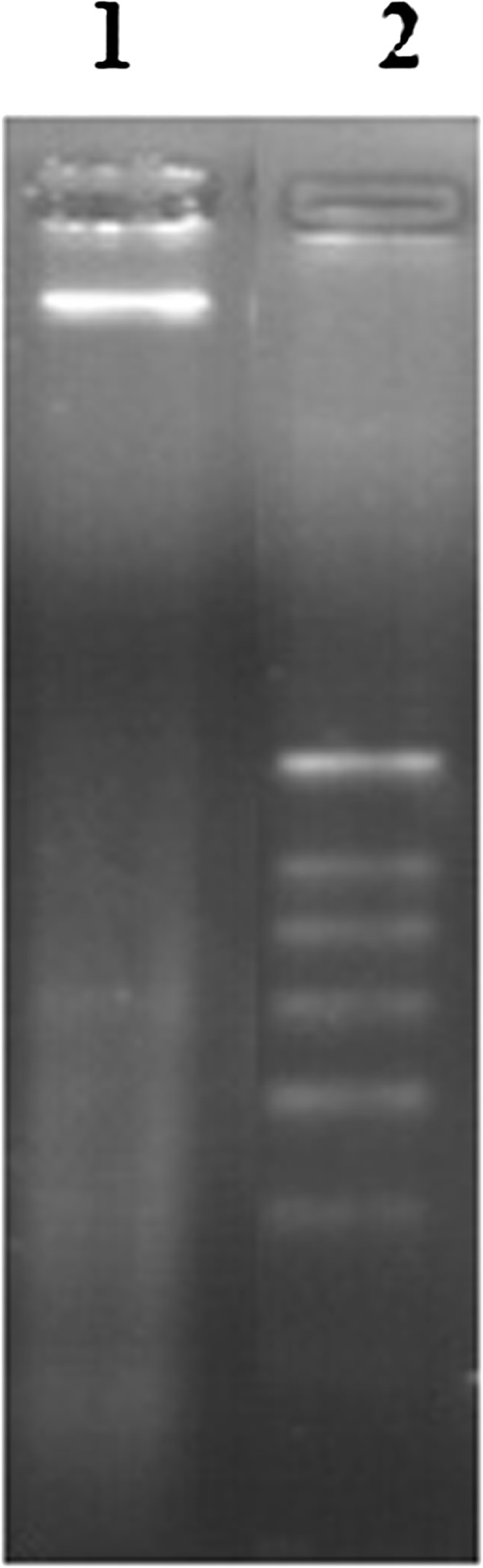
**Agarose gel electrophoresis shows DNA fragmentation indicating induction of apoptosis by the AEFLL in** ***HEp 2*** **cells.** Lane 1: treated with 200 μg/mL, lane 2: DNA molecular weight marker.

DNA fragmentation was observed at a concentration of 200 μg/mL of AEFLL after 24 hour exposure (Figure 4).

## Discussion

Phenolics compounds are secondary metabolites in plants, which are very important for their essential functions in reproduction and growth, in defense mechanisms, for its survival against pathogens, parasites, predators and solar radiation [24, 25]. Phenolic compounds also provide us natural antioxidants for protection against many diseases.

It has been determined that the antioxidant effect of plant products is mainly due to the radical scavenging activity of phenolic compounds such as flavonoids, polyphenols, tannins, and phenolic terpenes. They have the capability to scavenge reactive oxygen species (ROS), which include radical and nonradical oxygen species such as O_2_^−.^, HO^.^, NO^.^, H_2_O_2_, HOCl, as well as oxidatively generated free radicals RO^.^ and ROO^.^, ONOO − which derive from biomolecules including lipoproteins (LDL), proteins, and oligonucleic acids [2, 25]. The total phenolic content (mean ± SD) of the AEFLL was 22.15 ± 1.65% of GAE. It is reported that the total phenolic content of the *F. leucopyrus* fruit was 31.7 ± 4.92 GAE//100 g and DPPH scavenging activity expressed in mg of ascorbic acid equivalent antioxidant capacity (AEAC) per 100 grams of fruit was 76.8 (14). ROS/RNS can cause DNA base changes, strand breaks, damage to tumour-suppressor genes and enhanced expression of protooncogenes (1). The capacity for nitric oxide scavenging activity by the AEFLL was found to be highest, followed by DPPH and hydroxyl radical scavenging activities respectively (Table 1). Therefore there might be a direct involvement by the AEFLL in the inhibition of DNA and lipid oxidation by peroxynitrite anion (ONOO^−^) which are generated by over production of nitric oxide and the superoxide anion.

Brine shrimp bioassay is considered as prescreening assay for cytotoxicity, various pharmacological actions and pesticidal effects and it determines the lethality of materials towards brine shrimp larvae. However it is not specific for antitumor activity (20). The AEFLL showed no single death of larvae over a concentration of 50–500 μg/mL for brine shrimp lethality bioassay.

The methods used to study the inhibition of cell proliferation were the MTT and LDH assays on *HEp-2* cells. The MTT and LDH assays are well-established methods to assess mitochondrial competence and cell membrane integrity, respectively [26]. As evident in Figure 1, concentration dependent increase was observed in cytotoxicity over a range of 100–600 μg/mL of AEFLL with an EC_50_ of 506.80 ± 72.93 μg/mL for the MTT assay. The percentage of LDH release for the AEFLL was increased steadily in *HEp-2* cells over the concentration range of 100-600 μg/mL with an EC_50_ of 254.52 ± 42.92 μg/mL. A maximum of 80% inhibition of cell proliferation was observed at concentrations over 600 μg/mL for both MTT and LDH assays. Interestingly there is a decline in LDH release in to the culture medium at concentrations over 700 μg/mL. This indicates that there is a reduction in cell density after treatment by the AEFLL at concentrations >700 μg/mL, and also closely associates with MTT results showing maximum inhibition at these concentrations. It is observed that at 100 μg/mL, the percentage leakage of LDH was 26.54% which is similar to that of the negative control (26.52 ± 1.69%,). This indicates that *F. leucopyrus* shows cytotoxicty on *HEp-2* cells over a concentration of 100 μg/mL after 24 hour exposure. Morphological changes and lower cell density observed with the concentration further supports the inhibition of cell proliferation. To determine whether the growth inhibitory activity of the AEFLL was related to the induction of apoptosis, the cells were investigated using the AO/EB double staining under fluorescence microscopy after 24 hour treatment by the plant extract. The fluorescent images show, the signs of early apoptosis (yellow) and late apoptotic cells (orange red). This is further confirmed by the DNA ladder pattern. A separate study has been carried out in Sri Lanka to evaluate cytotoxicity of the aqueous extract of leaves by comet assay after exposure to whole blood (20 μl). Fragmented DNA (comets) has been observed at a concentration of 20 μg/ml but not at 05 μg/ml [27]. Phytochemicals gallic acid quercetin, kaempferol, coumarin which are reported previously as some of the constituents in the twigs and leaves of *F. leucopyra* have shown *in vitro* growth inhibition of breast, prostate, ovarian, liver cancer cells and acute leukemias [16, 28–36]. It is reported that dietary polyphenols inhibit key signal transduction protein kinases, such as mitogen activated protein kinase, and certain cyclin-dependent kinases which are necessary for cell growth and transformation (4). The contribution of penolics present in *F leucopyrus* needs further investigations to explore the mechanisms of inhibition related to cell proliferation and induction of apoptosis.

## Conclusion

*F. leucopyra* is considered as a plant containing anticancer activity and the water extract of leaf is consumed as a dietary supplement. The high antioxidant activity and phenolic content shown by the aqueous extract of the plant suggest that it is a potential therapeutic agent for the control of oxidative damage caused by reactive oxygen species and especially nitrogen species. *F. leucopyra* showed DNA fragmentation in *HEp2* cells even after 24 hour exposure of the leaf extract indicating its ability to induce apoptosis. This study provides the scientific proof of the traditional knowledge in using the leaf extract as an anticancer agent.

## Abbreviations

AEFLL: Aqueous extract of *F. leucopyrus* leaves
AO/EB: Acridine orange (AO)/ ethidium bromide (EB)
GAE: Gallic acid equivalents
DPPH: 1-Diphenyl-2-picrylhydrazyl
MTT: (3 4, 5-(dimethylthiazol-2-yl) 2-5-diphenyl tetrazolium bromide)
LDH: Lactate dehydrogenase.
